# Significantly Increased Risk of All-Cause Mortality Among Type 2 Diabetes Patients Living Alone

**DOI:** 10.3389/fmed.2022.782751

**Published:** 2022-01-26

**Authors:** Liyao Fu, Ying Zhou, Jiaxing Sun, Zhenhua Xing, Yongjun Wang, Shi Tai

**Affiliations:** ^1^Department of Blood Transfusion, The Second Xiangya Hospital of Central South University, Changsha, China; ^2^Department of Cardiovascular Medicine, The Second Xiangya Hospital of Central South University, Changsha, China

**Keywords:** type 2 diabetes, living alone, all-cause mortality, hazard ratio, congestive heart failure (CHF), coronary heart disease (CAD)

## Abstract

**Background:**

There is a lack of studies evaluating the association between living status and subsequent outcomes in patients with type 2 diabetes (T2DM).

**Objectives:**

This study aimed to assess the association between living alone and the risk of all-cause mortality in T2DM patients.

**Methods:**

We performed a secondary analysis in patients with long-lasting T2DM from the Action to Control Cardiovascular Risk in Diabetes (ACCORD) study. The primary outcome was all-cause mortality. Multivariable Cox proportional hazard models was used to analyze and compare the hazard ratios (HRs) in patients living alone and with one or more adults.

**Results:**

This study included 10,249 patients with T2DM. Of these, 2,078 (20.28%) were living alone and 8,171 (79.72%) lived with one or more adults. Over a median total follow-up of 8.8 years, 1,958 patients developed the primary endpoint. The all-cause mortality rates in patients living alone or living with one or more adults were 23.24 and 18.05%, respectively. Cox proportional hazard analysis showed that T2DM patients living alone had significantly higher rate of all-cause mortality than those living with others (HR, 1.34; 95% confidence interval [CI], 1.20–1.48; *p* < 0.001). After multivariable adjustment, living alone was an independent risk factor for all-cause mortality in patients with T2DM (adjusted HR, 1.27; 95% CI, 1.14–1.41; *p* < 0.001). Furthermore, the risks of both congestive heart failure (CHF) and fatal coronary heart disease (CHD) among 4,050 propensity score-matched patients were higher for patients living alone (respectively HR, 1.37; 95% CI, 1.08–1.74; *p* = 0.010; and HR, 1.16; 95% CI, 1.00–1.34; *p* = 0.047).

**Conclusions:**

The risk of all-cause mortality was significantly higher in T2DM patients living alone than in those living with one or more adults.

## Introduction

The number of individuals living alone is increasing among older people in developing and developed countries, and this is considered an important demographic and social change (1). In 2017, 33.6% of the households in the European Union and around 40% of those in Nordic countries (except Iceland) consisted of one individual living alone (2). Complex reasons explain this trend, such as, for instance, trends toward longevity, high divorce rates, high rates of widowhood, and low rates of intergenerational co-residence (3). Living alone may cause social isolation and feelings of loneliness and depression, especially when individuals perceive that their social needs are not met. The influence of social isolation on mortality and morbidity has been established among the traditional clinical risk factors (2, 4, 5). Meanwhile, loneliness and depression can negatively impact health and survival. Meta-analytic evidence demonstrates that loneliness is a predictor of all-cause mortality, showing that lonely people have a 22% higher risk of death than do non-lonely people (1). Moreover, living alone also entails a higher cost of living and may increase the economic burden of low-income people (6). Thus, living alone arises as a new concern with aging in patients with chronic non-communicable diseases.

Previous studies have demonstrated a significant association between living alone and mortality among older people (7, 8). The causal pathways connecting living alone with mortality are multifactorial. The social networks of individuals living alone tend to shrink, and these individuals are also likely to be in poorer health. Meanwhile, patients living alone have an increasing trend toward poor health behaviors (5, 9), and are also more likely to experience unmet care needs (10). In addition, several studies found that single living increased worse outcomes post heart attack or myocardial infarction (11, 12). More recently, our previous study demonstrated living alone is an independent risk factor for 1-year all-mortality in acute coronary syndrome patients ≥75 years of age (13). Given type 2 diabetes mellitus (T2DM) has been associated with the onset of atherosclerotic cardiovascular disease among older patients, often presenting as coronary heart disease (CHD), cerebrovascular disease, and cardiovascular death of atherosclerotic origin in patients (14), subsequently promoting premature aging. Therefore, there is an urgent need for cardiovascular events prevention in diabetic individuals. To achieve this goal, it is necessary to identify specific high-risk factors affecting the prognosis of T2DM in primary care.

The percentage of T2DM patients living alone has been reported to be ~7–15% (15). To date, no study has prospectively assessed the association between living alone and incident T2DM, although cross-sectional studies have investigated living alone as a risk factor for T2DM (16, 17). As individuals with T2DM tend to live for a long time with advanced comorbidities, it is significant for public health to determine whether living status is independently associated with poor clinical outcomes. However, previous studies have exclusively focused on the relationship between living alone and the incidence of T2DM. Therefore, the present study examined the association between living alone and clinical outcomes in T2DM. We used the data from the Action to Control Cardiovascular Risk in Diabetes (ACCORD) study (18) and the ACCORD Follow-On Study (ACCORDION) (19) to assess the association between living arrangements and all-cause mortality in patients with T2DM.

## Methods

### Study Participants and Data Collection

We performed a *post-hoc* analysis of the data from the ACCORD trial (ClinicalTrials.gov number, NCT00000620; data obtained from the Biologic Specimen and Data Repository Information Coordinating Center, National Heart, Lung and Blood Institute, U.S. Department of Health and Human Services). The rationale and design of the ACCORD trial have been described previously (20). Briefly, the ACCORD trial was a 2 × 2 factorial trial managed at 77 clinical sites in the United States and Canada, which recruited 10,251 T2DM patients aged between 40 and 79 years with glycosylated hemoglobin (HbA1c) concentration of 7.5% or more. The trial was designed to test whether the intensified control of blood glucose, blood pressure, and lipids could reduce the incidence of cardiovascular disease (CVD) in patients with T2DM. The included patients had a history of CVD, indicated by anatomical evidence of significant atherosclerosis, albuminuria, left ventricular hypertrophy, or at least two risk factors for cardiovascular diseases. Intensive control of blood pressure and lipids did not reduce CVD. However, intensive glycemic intervention was discontinued after a mean follow-up of 3.7 years because of the increased mortality in the intensive glycemic control group, and all participants were transitioned to standard glycemic control intervention. The ACCORD closeout visits were completed in June of 2009. Follow-up continued for the remaining participants in the ACCORDION trial, with a total follow-up period of 8.8 years. Ethics approval and consent to participate were not applicable.

### Exposure Variables

We excluded participants whose living arrangement baseline data were missing (*n* = 2). This resulted in a final sample of 10,249 participants for the analysis of the association between baseline living status and clinical outcomes. Living arrangement status at baseline was documented as either living alone or living with one or more adults. Further information collected at baseline included demographics, medical history, previous cardiovascular events, mental health, laboratory values (e.g., fasting blood glucose, HbA1c, estimated glomerular filtration rate (eGFR), total cholesterol, and triglycerides), and current chronic drug regimen.

### Study Outcomes and Definitions

The primary outcome of this study was all-cause mortality. Secondary endpoints were cardiovascular mortality, non-fatal stroke, non-fatal myocardial infarction (MI), congestive heart failure (CHF), and fatal coronary heart disease (CHD). Patients were followed up every 2–4 months through phone interviews or visits at the outpatient clinic. At 4-month intervals, the relevant medical information was collected. The study outcomes were classified by the Working Group of the Morbidity and Mortality subcommittee.

### Statistical Analysis

Qualitative demographic data are presented as numbers (percentages), and baseline characteristics of patients living alone and living with others were compared using the chi-square test. Quantitative data are presented as mean ± SD, and the Student's *t*-test was used to compare baseline characteristics. Kaplan-Meier survival curves were used to analyze primary and secondary outcomes in patients living alone or living with others, and the differences between groups in cumulative incidence curves were compared using the log-rank test. A Cox proportional hazards regression model was used to calculate the hazard ratio (HR) and 95% confidence intervals (CIs) for the primary and secondary outcomes in the comparisons of patients living alone or living with others. The proportional hazards assumption was examined using Schoenfeld residuals. Three multivariable models with progressive degrees of adjustment were used to adjust for potential confounders of the study outcomes. Model 1 was adjusted for age, sex, race, body mass index (BMI), previous cardiovascular events, education level, systolic blood pressure (SBP), diastolic blood pressure (DBP), and smoking status. Model 2 was further adjusted for other clinical variables, including duration of diabetes, eGFR, HbA1c, total plasma cholesterol, plasma high-density lipoprotein cholesterol (HDL-C), plasma low-density lipoprotein cholesterol (LDL-C), and depression status. Model 3 was further adjusted for the use of statins, biguanide, aspirin, angiotensin-converting enzyme inhibitor/angiotensin receptor blocker (ACEI/ARB), and insulin.

The primary and secondary outcomes in propensity score-matched patients with different living statuses were determined using Cox proportional hazard analysis. We used 1:1 nearest-neighbor matching without replacement to match all the baseline characteristics. The propensity score was calculated using a logistic regression model. Standardized differences <0.10 between propensity score-matched patients were considered negligible. The effect of living alone in patients with T2DM was further analyzed according to subgroup analysis: sex (male or female), age (<60 or ≥60 years), race (white or non-white), CVD (CVD history or no CVD history), HbAc1 level (<8.0% or ≥8.0%), depression (depression or non-depression), smoking (no history of smoking or history of smoking), and use of insulin or statins. Statistical significance was set at *p* < 0.05. All statistical analyses were performed using the Statistical Product and Service Solution version 25 (IBM, Armonk, NY, USA).

## Results

### Baseline Characteristics According to Living Arrangement

A total of 10,249 patients were eligible for inclusion in this analysis, including 2,078 documented as living alone (20.28%) and 8,171 (79.72%) living with one or more adults. Patients enrolled in the current study were 62.76 ± 6.64 years old on average. The baseline characteristics are shown in Table 1. Participants living alone were older and more often female and white. They had higher BMI, heart rate (HR), HbAc1, total cholesterol, LDL-C, urinary creatinine, urinary albumin, and lower levels of eGFR than those living with one or more adults (all *p* < 0.001). Likewise, participants living alone had more frequent smoking history, higher prevalence of CVD, prior hospitalization for heart failure (HF), depression, and CHF, were more prone to taking metformin and insulin, and less prone to take statins than patients living with one or more adults (all *p* < 0.001).

**Table 1:** Characteristics of patients with different living status.

**Variable**	**All (*n* = 10,249)**	**Living alone**		***P*-value**
		**No (*n* = 8,171)**	**Yes (*n* = 2,078)**	
Age (year; mean ± SD)	62.76 ± 6.64	62.54 ± 6.58	63.66 ± 6.79	<0.001
**Sex no. (%)**				
Male	6,299 (61.46%)	5,323 (65.15%)	976(46.97%)	<0.001
Female	3,950 (38.54%)	2,848 (34.85%)	1,102 (53.03%)	
**Race no. (%)**				<0.001
White	6,392 (62.37%)	5,177 (63.35%)	1,215 (58.47%)	
Non-white	3,857 (37.63%)	2,994 (36.64%)	863 (41.53%)	
Median duration of diabetes (year; mean ± SD)	10.80 ± 7.60	10.77 ± 7.52	10.93 ± 7.89	0.407
Median duration of hyperlipidemia (year; mean ± SD)	5.96 ± 5.70	5.95 ± 5.66	5.96 ± 5.86	0.985
Median duration of hypertension (year; mean ± SD)	10.23 ± 9.58	10.12 ± 9.45	10.67 ± 10.09	0.049
Previous cardiovascular events no. (%)	3,608 (35.20%)	2,942 (36.01%)	666 (32.05%)	0.001
**Smoking status no. (%)**				0.387
No smoking	4,294 (41.09%)	3,406 (41.68%)	888 (42.73%)	
Smoking	5,955 (58.0%)	4,765 (58.32%)	1,190 (57.27%)	
**Education no. (%)**				
Less than high school graduate	1,521 (14.84%)	1,219 (14.92%)	302 (14.53%)	0.665
High school graduate	2,704 (26.38%)	2,169 (26.55%)	535 (25.75%)	0.467
Some college or technical school	3,357 (32.75%)	2,653 (32.47%)	704 (33.88%)	0.216
College degree or higher	2,661 (25.96%)	2,126 (26.02%)	535 (25.75%)	0.809
Previous heart failure no. (%)	494 (4.82%)	386 (4.72%)	108 (5.02%)	0.369
Depression no. (%)	2,419 (23.60%)	1,797 (21.99%)	622 (25.71%)	0.000
Heart rate (mean ± SD)	72.65 ± 11.82	72.34 ± 11.64	73.88 ± 12.43	<0.001
SBP (mmHg, mean ± SD)	136.36 ± 17.11	136.31 ± 16.97	136.56 ± 17.66	0.564
DBP (mmHg, mean ± SD)	74.89 ± 10.58	74.89 ± 10.58	74.83 ± 10.96	0.822
BMI (mean ± SD)	32.22 ± 5.42	30.10 ± 5.38	32.68 ± 5.57	<0.001
Glycated hemoglobin (%, mean ± SD)	8.30 ± 1.06	8.2 ± 1.05	8.4 ± 1.07	0.011
eGFR (mL/min, mean ± SD)	91.05 ± 27.15	91.48 ± 27.50	89.36 ± 56.70	0.001
FPG (mg/dL, mean ± SD)	175.19 ± 56.18	174.7 ± 55.79	177.12 ± 57.66	0.087
ALT (U/L, mean ± SD)	27.58 ± 16.19	27.97 ± 16.68	26.05 ± 13.98	<0.001
Potassium (mg/dL, mean ± SD)	4.48 ± 0.47	4.48 ± 0.47	4.46 ± 0.49	0.194
Cholesterol (mg/dL, mean ± SD)	183.29 ± 41.85	182.45 ± 41.69	186.56 ± 42.50	0.001
Triglyceride (mg/dL, mean ± SD)	190.13 ± 148.40	190.5 ± 143.81	188.26 ± 165.30	0.559
Low-density lipoprotein (mg/dL, mean ± SD)	104.89 ± 33.93	104.37 ± 33.67	106.97 ± 34.79	0.002
High-density lipoprotein (mg/dL, mean ± SD)	41.86 ± 11.62	41.41 ± 11.25	43.63 ± 12.79	<0.001
Serum creatinine (mg/dL, mean ± SD)	0.91 ± 0.23	0.91 ± 0.23	0.91 ± 0.24	0.883
Urinary albumin (mg/dL, mean ± SD)	10.27 ± 36.60	9.77 ± 34.75	12.23 ± 42.08	0.017
Urinary creatinine (mg/dL, mean ± SD)	124.41 ± 66.25	123.10 ± 65.16	129.54 ± 70.14	<0.001
**Medications no. (%)**				
Insulin	3,581 (34.94%)	2,816 (34.46%)	765 (36.81%)	0.045
Metformin	6,553 (63.94%)	5,277 (64.58%)	1,276 (61.41%)	0.007
ACEI/ARB	7,100 (69.28%)	5,662 (69.29%)	1,438 (69.20%)	0.935
Statin	6,499 (63.41%)	5,238 (64.10%)	1,261 (60.68%)	0.004
Aspirin	5,579 (54.43%)	4,456 (54.53%)	1,123 (54.04%)	0.688
MMSE score (mean ± SD)	27.40 ± 2.51	27.39 ± 2.52	27.44 ± 2.47	0.634
All-cause mortality	1,958 (19.10%)	1,475 (18.05%)	483 (23.24%)	<0.001

*Values are mean ± SD or %. DBP, diastolic blood pressure; SBP, systolic blood pressure; BMI, body mass index; eGFR, estimated glomerular filtration rate; FPG, fasting plasma glucose; ALT, Alanine aminotransferase; MMSE, mini-mental State Examination; ACEI, angiotensin-converting enzyme inhibitors; ARB, angiotensin II receptor blockers; COPD, chronic obstructive pulmonary disease; MI, myocardial infarction*.

### Association Between Living Arrangement and All-Cause Mortality

**Figure 1:**
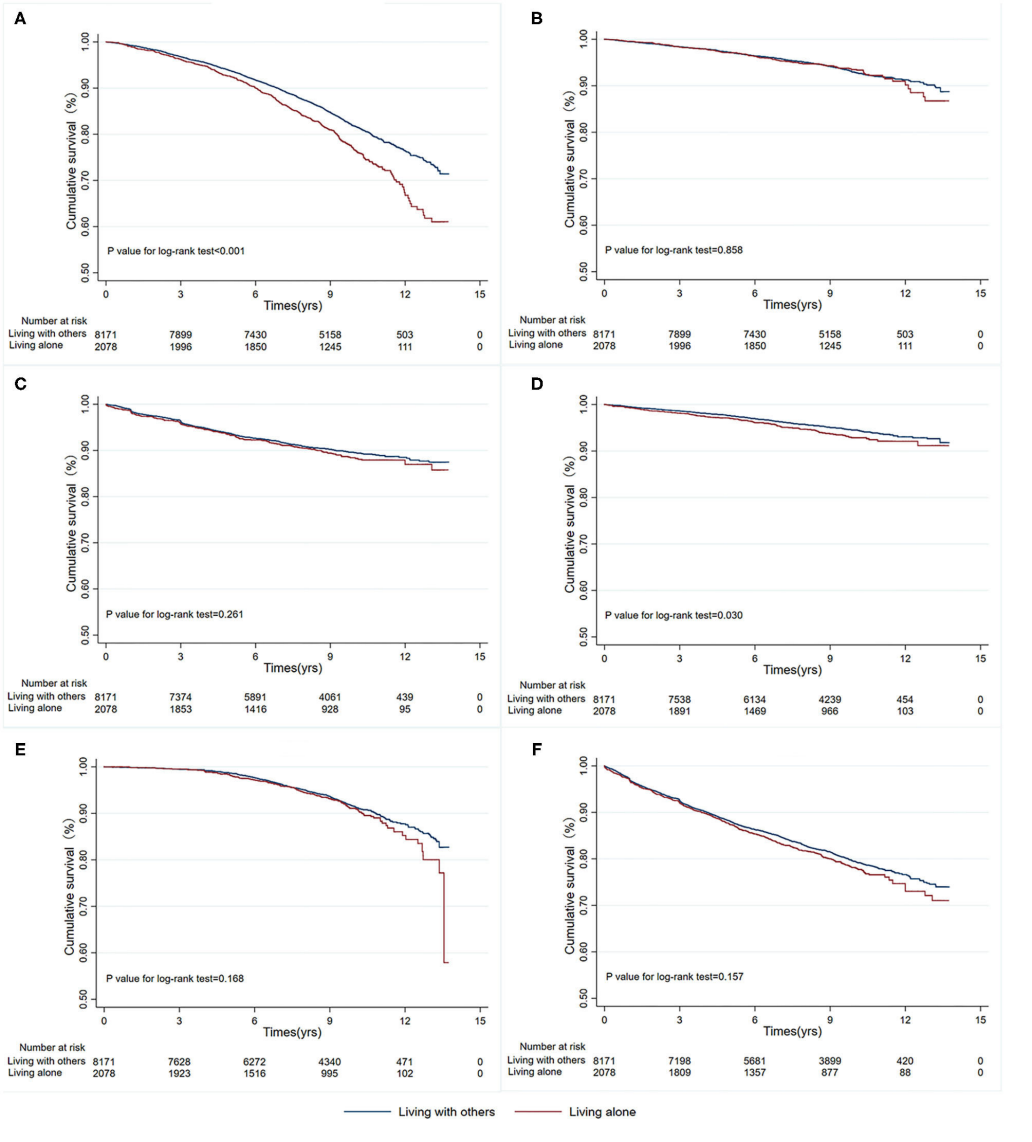
Kaplan-Meier survival curves for primary and secondary outcomes by living status. **(A)** All-cause mortality; **(B)** Cardiovascular mortality; **(C)** Non-fatal MI; **(D)** Non-fatal stroke; **(E)** CHF; **(F)** CHD. MI, myocardial infarction; CHF, congestive heart failure; CHD, coronary artery disease.

During a median follow-up of 8.8 years, 1,958 patients (19.10%) developed all-cause mortality. As Table 2 shows, the incidence of all-cause mortality was higher in patients who lived alone than in those living with other adults (483 [23.24%] vs. 1,475 [18.05%], *P* = 0.001). In the unadjusted model, patients living alone had a higher risk of all-cause mortality (HR, 1.34; 95% CI, 1.20-1.48; *p* < 0.001) and non-fatal stroke (HR, 1.26; 95% CI, 1.02-1.56; *P* = 0.030) than those living with one or more adults. There was no difference in the rates of cardiovascular mortality, non-fatal MI, CHF, or CHD. Kaplan-Meier survival curves and cumulative event rates for the primary and secondary outcomes in patients with different living statuses are shown in Figure 1 and Table 2, respectively. In the multivariable model, there remained statistically significant differences in all-cause mortality (model 1: adjusted HR, 1.31; 95% CI, 1.18–1.46; *p* < 0.001; model 2: adjusted HR, 1.27; 95% CI, 1.14–1.41; *p* < 0.001; model 3: adjusted HR, 1.27; 95% CI, 1.14–1.41; *p* < 0.001). There were no differences between patients living alone and those living with one or more adults in cardiovascular mortality, non-fatal MI, non-fatal stroke, CHF, and CHD.

**Table 2:** The risk of primary and second outcomes in T2DM Patients with different living status.

**Characteristics**	**Living with others**	**Living alone**	***p*-value**
**All-cause mortality**			
Cases/*n*	1,475/8,171	483/2,078	
Unadjusted HR (95% CI)	1.00 (ref)	1.34 (1.20–1.48)	<0.001
Model 1: adjusted HR (95% CI)	1.00 (ref)	1.31 (1.18–1.46)	<0.001
Model 2: adjusted HR (95% CI)	1.00 (ref)	1.27 (1.14–1.41)	<0.001
Model 3: adjusted HR (95% CI)	1.00 (ref)	1.27 (1.14–1.41)	<0.001
**Cardiovascular mortality**			
Cases/*n*	535/8,171	134/2,078	
Unadjusted HR (95% CI)	1.00 (ref)	1.02 (0.84–1.23)	0.858
Model 1: adjusted HR (95% CI)	1.00 (ref)	1.03 (0.85–1.25)	0.782
Model 2: adjusted HR (95% CI)	1.00 (ref)	1.02 (0.83–1.24)	0.871
Model 3: adjusted HR (95% CI)	1.00 (ref)	1.02 (0.83–1.24)	0.881
**Non-fatal MI**			
Cases/n	738/8,171	198/2,078	
Unadjusted HR (95% CI)	1.00 (ref)	1.09 (0.94–1.28)	0.261
Model 1: adjusted HR (95% CI)	1.00 (ref)	1.13 (0.96–1.33)	0.130
Model 2: adjusted HR (95% CI)	1.00 (ref)	1.08 (0.92–1.27)	0.359
Model 3: adjusted HR (95% CI)	1.00 (ref)	1.08 (0.92–1.27)	0.349
**Non-fatal stroke**			
Cases/n	374/8,171	114/2,078	
Unadjusted HR (95% CI)	1.00 (ref)	1.26 (1.02–1.56)	0.030
Model 1: adjusted HR (95% CI)	1.00 (ref)	1.25 (1.01–1.55)	0.042
Model 2: adjusted HR (95% CI)	1.00 (ref)	1.18 (0.94–1.46)	0.149
Model 3: adjusted HR (95% CI)	1.00 (ref)	1.18 (0.95–1.47)	0.145
**CHF**			
Cases/n	549/8,171	147/2,078	
Unadjusted HR (95% CI)	1.00 (ref)	1.14 (0.95–1.36)	0.168
Model 1: adjusted HR (95% CI)	1.00 (ref)	1.10 (0.91–1.33)	0.306
Model 2: adjusted HR (95% CI)	1.00 (ref)	1.08 (0.89–1.30)	0.446
Model 3: adjusted HR (95% CI)	1.00 (ref)	1.07 (0.89–1.30)	0.468
**CHD**			
Cases/*n*	1,471/8,171	388/2,078	
Unadjusted HR (95% CI)	1.00 (ref)	1.08 (0.97–1.21)	0.157
Model 1: adjusted HR (95% CI)	1.00 (ref)	1.14 (1.01–1.28)	0.027
Model 2: adjusted HR (95% CI)	1.00 (ref)	1.11 (0.98–1.24)	0.091
Model 3: adjusted HR (95% CI)	1.00 (ref)	1.11 (0.99–1.24)	0.082

*Model 1, the following parameters were adjusted: age, sex, previous cardiovascular events, race, BMI, education, SBP, DBP, and smoking status.*

*Model 2, the following parameters were adjusted: age, sex, previous cardiovascular events, race, education, duration of diabetes, SBP, DBP, smoking status, eGFR, HbA1c, total plasma cholesterol, plasma HDL-C, plasma LDL-C, and depression.*

*Model 3, the following parameters were adjusted: age, sex, previous cardiovascular events, race, BMI, education, duration of diabetes, depression, SBP, DBP, smoking status, eGFR, HbA1c, total plasma cholesterol, plasma HDL-C, plasma LDL-C, use of statin or biguanide, aspirin, ACEI/ARB, and insulin*.

**Figure 2:**
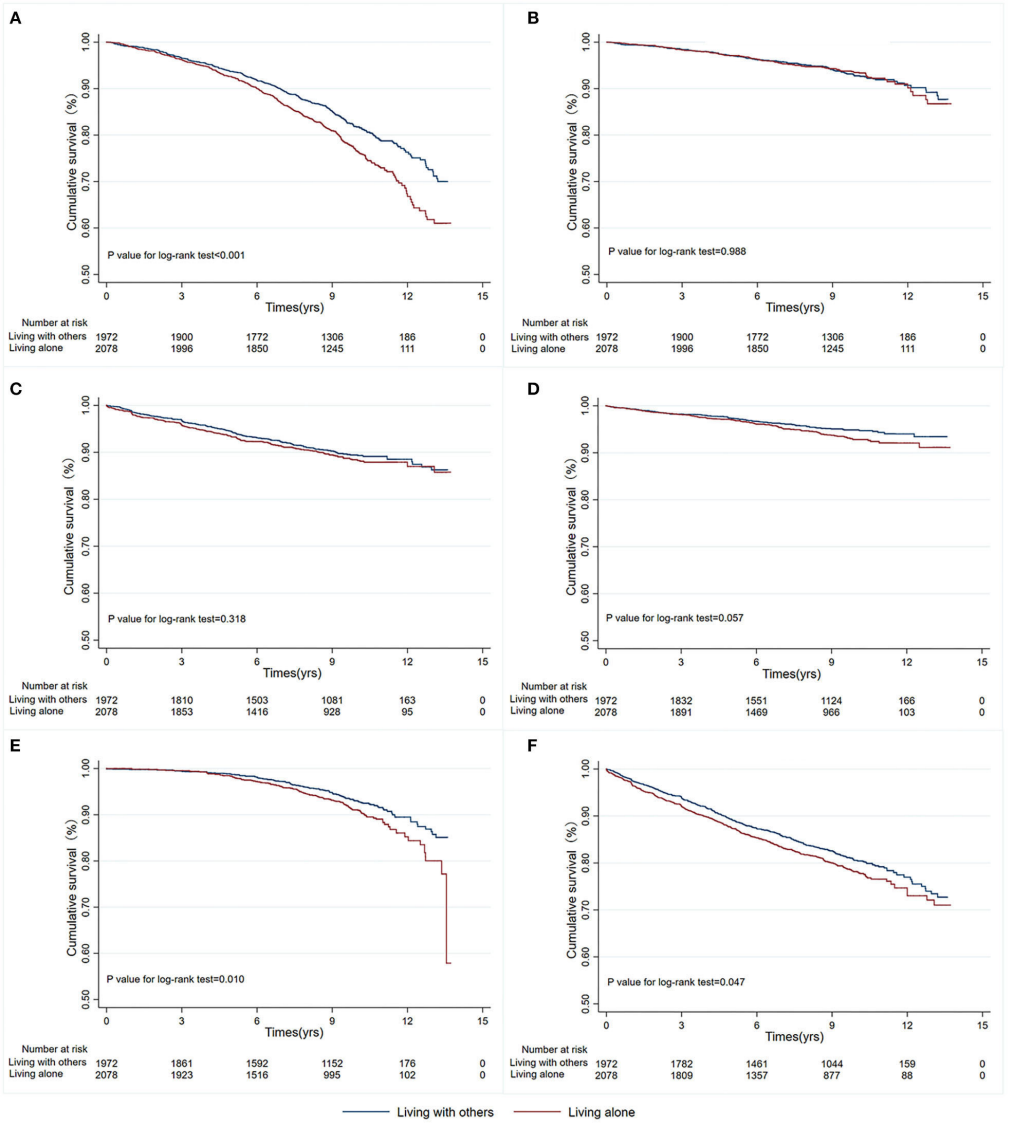
Kaplan-Meier survival curves for primary and secondary outcomes in propensity score-matched patients with different living status. **(A)** All-cause mortality; **(B)** Cardiovascular mortality; **(C)** Non-fatal MI; **(D)** Non-fatal stroke; **(E)** CHF; **(F)** CHD. MI, myocardial infarction; CHF, congestive heart failure; CHD, coronary artery disease.

We used propensity score matching as a sensitivity analysis to verify the association between living alone and the risk of primary and secondary outcomes in patients with T2DM. Among the propensity score-matched patients (n=4,050), the risk of all-cause mortality (HR, 1.34; 95% CI, 1.17–1.53; *P*< *0*.001), CHF (HR, 1.37; 95% CI, 1.08–1.74; *P* = 0.010), and CHD (HR, 1.16; 95% CI, 1.00–1.34; *P* = 0.047) were significantly higher in patients living alone than in those living with one or more adults, whereas there were no differences in the risk of adverse CV events, cardiovascular mortality, non-fatal stroke, and non-fatal MI. Kaplan-Meier survival curves and cumulative event rates for primary and secondary outcomes are shown in Figure 2.

### Association Between Living Arrangements and All-Cause Mortality in Different Subgroups

**Figure 3:**
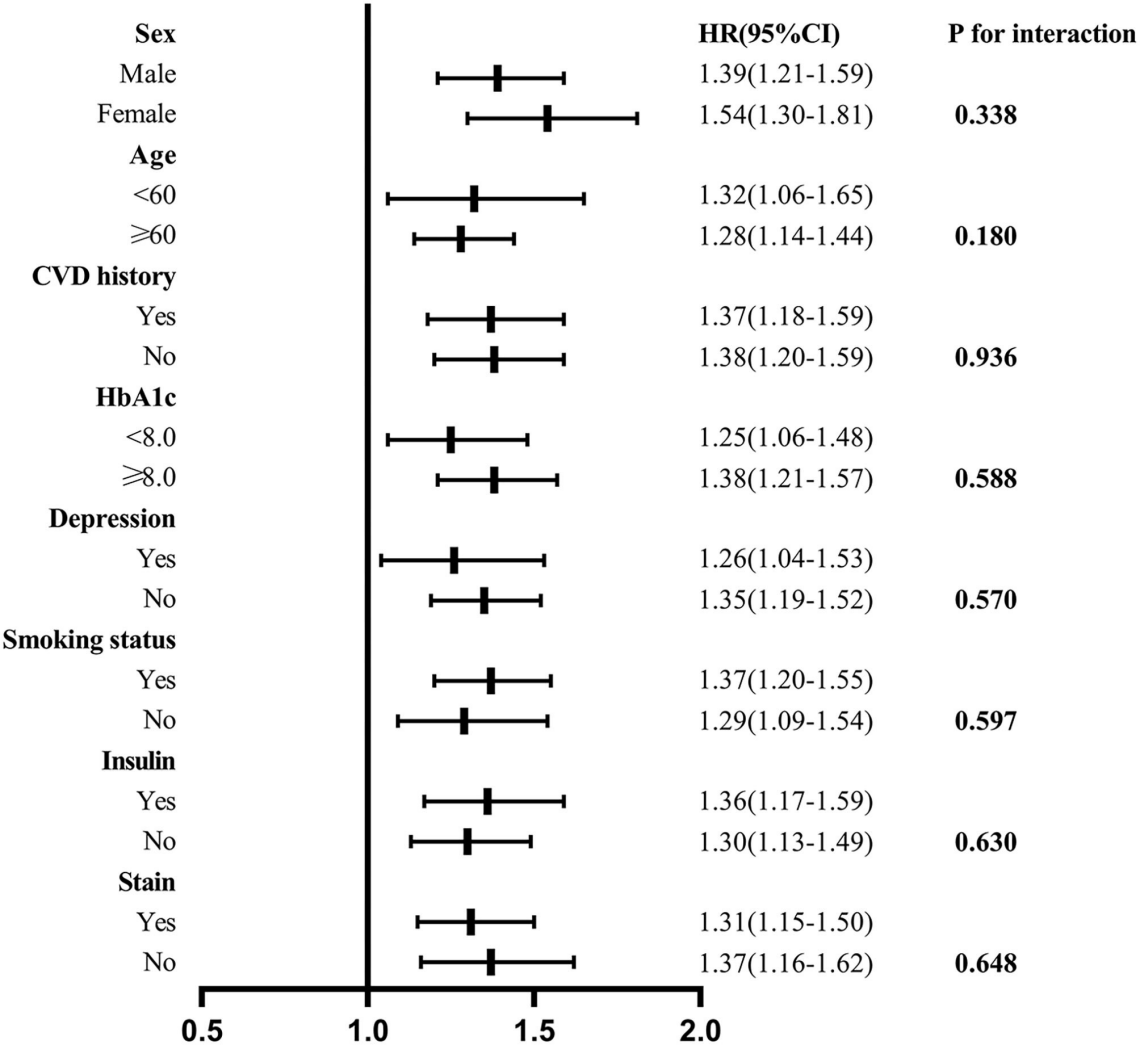
Association between living alone and all-cause mortality in the subgroups. Interaction and stratified analyses were performed to evaluate the association between living arrangements and all-cause mortality in the different subgroups.

Interaction and stratified analyses were performed to evaluate the association between living arrangements and all-cause mortality in the different subgroups (Figure 3). We did not find interactions among age, sex, previous history of CVD, depression status, smoking history, HbA1C, use of insulin, or use of statins, suggesting that the results of different subgroups are consistent and reliable.

## Discussion

In this study, we found an association between living alone and mortality in T2DM. Unadjusted analysis showed that people living alone had a higher incidence of all-cause mortality (the primary endpoint) and non-fatal stroke. However, there were no differences in CV mortality, non-fatal MI, CHF, or CHD between the two groups. Importantly, living alone in patients with T2DM was independently associated with an increased risk of all-cause mortality after adjusting for confounding variables. However, the adjusted analysis revealed that living alone was not an independent predictor of non-fatal stroke. These results highlight the clinical importance of living status in individuals with T2DM. The association between living alone and increased risk of all-cause mortality was observed among the prespecified subgroups. This phenomenon could be significant for public health in consideration of the increasing incidence of living alone and how society as a whole and its healthcare systems adapt to this transformation.

The incidence of living alone continues to grow in the general population, and the present study showed that 20.28% of the ACCORD participants were living alone. Living status has been suggested as a risk factor for T2DM. T2DM patients are diverse in terms of ethnicity, life behaviors, socioeconomic status, and psychosocial factors that may play a role in the prognosis of T2DM. Previous studies revealed that the association between living alone and mortality persisted significantly, even after controlling for confounding variables (21). However, few studies have investigated the relationship between living alone and prognosis in patients with T2DM. Hence, it is necessary to evaluate the relationship between living status and adverse events in T2DM patients.

The present study demonstrated that T2DM patients living alone were characterized by older age, higher prevalence of cardiovascular events, higher prevalence of smoking habits, higher BMI, and higher levels of total cholesterol and LDL-C than those living with others. These findings suggest that multifactorial lifestyle modification interventions are likely to be effective in improving the prognosis of T2DM patients living alone. Moreover, our results showed that patients living alone had lower quality of life, suggesting a critical need for adjusting treatment and management strategies to improve the quality of life of these patients.

Our results expanded previous understanding and confirmed that living alone is an independent risk factor for the prognosis of T2DM patients in a long-term follow-up. However, the potential mechanisms underlying such association are unclear, several factors have been found to be associated with mortality. Several studies have found that individuals living alone have worse self-perceived health and quality of life, depression, and feelings of loneliness (22–24). Consistent with previous reports, our study further confirmed that T2DM patients living alone experienced more feelings of depression (25.71% vs. 21.99%, *p* < 0.001). Moreover, numerous studies have shown that depression is associated with a higher mortality rate (25). Katon et al. conducted a study on 4,000 patients with T2DM. Over a 3-year follow-up, the mortality in patients with mild or severe depression was 1.7 and 2.3 times higher, respectively, than that of patients without depression (26). Similar findings were reported by Zhang et al. in a survival analysis using the National Health and Nutrition Examination Survey (NHANES)-I data (27).

The majority of studies have also found that living alone is associated with poor diabetes self-care, and especially poor dietary arrangements (28). Dietary patterns are closely related to the optimal management of T2DM in the general population (28–30). The diverse features of people living alone and complex social and demographic changes could influence the dietary patterns of patients with T2DM. All these factors could affect compliance with the guidelines to optimize nutritional status. The relationship between living alone and dietary patterns has also been discussed previously (31, 32). Although a few studies found some healthy behaviors in patients living alone, most studies found that a larger number of patients living alone are less likely to follow healthy dietary habits, including the intake of diverse foods and the consumption of fruits and vegetables (31, 33). Furthermore, there is a possibility that a decline in motivation and pleasure in cooking and/or eating in people living alone, which often manifests in the cooking of simple meals or the consumption of ready-made food. The likely consequences are difficulty in following healthy eating recommendations and in controlling portion size. Aspects of psychological and mental health related to living alone could also affect food intake, resulting in increased or decreased dietary intake.

Previous studies have found that T2DM patients living alone show poor medication adherence, including to prescribed medications and blood measurements. Strict glycemic management is associated with a decreased risk of diabetes-related complications, especially in individuals who have not suffered years of uncontrolled HbA1c levels (34). Projections from the observational United Kingdom Prospective Diabetes Research (UKPDS 35) proposed that a 1 percent decrease in mean HbA1c would lead to a 14% lower risk of all-cause mortality, 21% lower rate of diabetes-related mortality, and 37% decline in the risk of microvascular complications (35). Spencer et al. suggested that education and support from peers allow T2DM patients to achieve better self-management in the long term, leading to good efficacy of HbA1c control (36). A link between living alone and worse HbA1c management has also been observed in a recent study (37). Our results are consistent with those previous findings, as the mean HbA1c level in T2DM patients living alone in our study was higher than that in patients living with one or more adults.

We also found that the risk of CHF and CHD was significantly higher in individuals living alone among propensity score-matched patients. Recent research has described the association between living alone and the incidence of adverse cardiovascular events (35, 38). The Coronary Revascularization Demonstrating Outcome Study in Kyoto of Acute Myocardial Infarction Registry (CREDO—Kyoto AMI) showed that, in a 5-year follow-up, individuals living alone had higher risk of admission for HF (39). The Reduction of Atherothrombosis for Continued Health (REACH) study also showed that living alone was associated with a higher risk of mortality and CV death (40). A possible explanation is that living alone may increase anxiety and depression, causing more psychological distress, poor handling mechanisms and self-care, less access to healthcare services, and less insistence on guideline-recommended therapy and secondary prevention targets.

## Limitations

The first limitation of this study is that it was a *post-hoc*, exploratory analysis of the ACCORD data; randomization may break, and residual and uncontrolled confounding may still be present. Additionally, the data included in the present study, derived from clinical trials, may not be representative of real-world populations of patients with T2DM. Third, we were unable to account or adjust for unidentified confounders, such as stress and socioeconomic status. Unfortunately, although the statistical modeling included multiple factors, including psychosocial factors and medical history, we acknowledge that there remains a potential for residual confounding. Fourth, living alone was assessed only once at baseline: we did not re-evaluate the living status during follow-up, during which cohabitation status or social circumstances may have changed. Information from prospective clinical trials is needed to clarify the practical effects of living alone in patients with T2DM.

## Conclusion

The present study suggests that living status may be a strong marker for predicting the prognosis of T2DM patients, an observation which warrants confirmation in further studies. The main significance of the present study was the identification of specific high-risk factors affecting the prognosis of T2DM. Therefore, these findings have potential implications for public health. Society as a whole needs to be prepared to the negative effects of the increasing rate of individuals living alone.

## Data Availability Statement

Publicly available datasets were analyzed in this study. The datasets are available from the ACCORD/ACCORDIN Research Materials obtained from the National Heart, Lung, and Blood Institute (NHLBI) Biologic Specimen and Data Repository Information Coordinating Center.

## Ethics Statement

The studies involving human participants were reviewed and approved by The Second Xiangya Hospital of Central South University. The patients/participants provided their written informed consent to participate in this study.

## Author Contributions

This study was completed in collaboration with the following authors: YW and ST defined the study theme and methods. LF, YZ, and JS analyzed the data. LF wrote the paper. ZX and ST edited the paper. All authors have read and approved the final manuscript.

## Funding

This research was supported by the National Natural Science Foundation of China (81801394 to ST) and the Natural Science Foundation of Hunan Province (2019JJ50878 to ST).

## Author Disclaimer

The contents of this report do not necessarily reflect the opinions or views of the ACCORD/ACCORDIN study authors or the NHLBI.

## Conflict of Interest

The authors declare that the research was conducted in the absence of any commercial or financial relationships that could be construed as a potential conflict of interest.

## Publisher's Note

All claims expressed in this article are solely those of the authors and do not necessarily represent those of their affiliated organizations, or those of the publisher, the editors and the reviewers. Any product that may be evaluated in this article, or claim that may be made by its manufacturer, is not guaranteed or endorsed by the publisher.
