# Supplementary material for: Significantly Increased Risk of All-Cause Mortality Among Type 2 Diabetes Patients Living Alone
Source: Front Med (Lausanne). 2022 Jan 26;9:782751. doi: 10.3389/fmed.2022.782751 (PMC8826243; doi:10.3389/fmed.2022.782751)
Supplement: Supplementary file 1 [file Table_1.DOCX]

**Supplementary Table 1. The health-related quality of life in T2DM patients with different living status**

| Variable |  | Living alone | | P value |
| --- | --- | --- | --- | --- |
|  | All (n=1963) | no（n= 1570） | yes（n=393） |  |
| PHQ-9 depression (mean ± SD) | 5.32±4.88 | 5.22±4.82 | 5.73±5.06 | 0.074 |
| SF-36 physical component score (mean ± SD) | -1.23±0.55 | -1.22±0.54 | -1.26±0.59 | 0.344 |
| SF-36 mental component score (mean ± SD) | 0.28±1.12 | 0.31±1.17 | 0.14±1.31 | 0.025 |
| Aggregate Interference Score (mean ± SD) | 0.68±0.08 | 0.69±0.08 | 0.67±0.09 | 0.013 |
| DTSQ treatment satisfaction scale (mean ± SD) | 73.55±9.74 | 74.05±19.67 | 71.55±20.00 | 0.029 |
| Diabetes Symptoms Distress Checklist (mean ± SD) | 59.90±43.14 | 59.40±43.31 | 61.89±42.45 | 0.302 |
